# Respiratory responses to hypercapnia and hypoxia in mice with genetic ablation of Kir5.1 (*Kcnj16*)

**DOI:** 10.1113/expphysiol.2010.055848

**Published:** 2011-01-14

**Authors:** Stefan Trapp, Stephen J Tucker, Alexander V Gourine

**Affiliations:** 1 Department of Surgery and Cancer, Biophysics Section, Imperial College LondonLondon, UK; 2 Clarendon Laboratory, Department of Physics, University of OxfordOxford, UK; 3 Neuroscience, Physiology & Pharmacology, University College LondonLondon, UK

## Abstract

Inward rectifier (Kir) potassium channels contribute to the control of electrical activity in excitable tissues and their activity is modulated by many biochemical factors, including protons. Heteromeric Kir4.1–Kir5.1 channels are highly pH sensitive within the physiological range of pH changes and are strongly expressed by the peripheral chemosensors as well as in the brainstem pH-sensitive areas which mediate respiratory responses to changes in blood and brain levels of 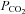/[H^+^]. In the present study, Kir5.1 knockout mice (Kir5.1^−/−^) were used to determine the role of these channels in the chemosensory control of breathing. We found that Kir5.1^−/−^ mice presented with persistent metabolic acidosis and a clear respiratory phenotype. Despite metabolic acidosis, ventilation at rest and in hyperoxic hypercapnia were similar in wild-type and Kir5.1^−/−^ mice. Ventilatory responses to hypoxia and normoxic hypercapnia were significantly reduced in Kir5.1^−/−^ mice; however, carotid body chemoafferent responses to hypoxia and CO_2_ were not affected. In the *in situ* brainstem–spinal cord preparations with denervated peripheral chemoreceptors, resting phrenic nerve activity and phrenic nerve responses to respiratory acidosis or isohydric hypercapnia were also similar in Kir5.1^−/−^ and wild-type mice. In *in situ* preparations of Kir5.1^−/−^ mice with intact peripheral chemoreceptors, application of CN^−^ resulted in a significantly reduced phrenic nerve response, suggesting that the relay of peripheral chemosensory information to the CNS is compromised. We suggest that this compensatory modulation of the peripheral chemosensory inputs develops in Kir5.1^−/−^ mice in order to counteract the effect of continuing metabolic acidosis on the activity of the peripheral chemoreceptors. These results therefore suggest that despite their intrinsic pH sensitivity, Kir4.1–Kir5.1 channels are dispensable for functional central and peripheral respiratory chemosensitivity.

Arterial CO_2_ drives respiratory activity in mammals by its actions on the peripheral respiratory chemoreceptors within the carotid and aortic bodies and central chemoreceptors located within the medulla oblongata and pons. The latter are responsible for up to 80% of the overall ventilatory response to CO_2_ (Heeringa *et al.* 1979), which is mediated by several populations of putative central respiratory chemoreceptor neurones identified within anatomically distinct brainstem regions, including locus coeruleus (LC), nucleus of the solitary tract (NTS), medullary raphe, retrotrapezoid nucleus (RTN) and others (Nattie & Li, 2009).

There is evidence that depolarization of central pH-sensitive neurones, including RTN neurones, is associated with a decreased K^+^ conductance (Mulkey *et al.* 2004). Two-pore domain K^+^ channels (TASKs) are highly sensitive to changes in extracellular pH within the physiological range (7.4–7.2) and have long been considered as the favoured molecular candidates to underlie chemosensory responses of the brainstem neurones to changes in pH. However, in mice, TASK-2 deficiency results in a complex respiratory phenotype with hypersensitivity to low levels of CO_2_ (Gestreau *et al.* 2010), while TASK-1 and TASK-3 were found to be dispensable for central respiratory chemosensitivity and, therefore, eliminated as the possible transducing molecules (Mulkey *et al.* 2007;Trapp *et al.* 2008).

A potential alternative mechanism could involve pH-sensitive K^+^ channels of the inward rectifier (Kir) family (Hibino *et al.* 2010). These channels contribute to the control of cellular activity in excitable tissues and their activity is influenced by many factors, including [H^+^]. Although Kir1.1, Kir2.3 and Kir4.1 are pH sensitive, their sensitivity lays outside the physiological range of pH changes in the brain (pK_a_ 6.8, 6.77 and 6.1, respectively) (Fakler *et al.* 1996; Zhu *et al.* 1999; Xu *et al.* 2000). Expression of Kir5.1 alone is unable to produce a functional channel (Bond *et al.* 1994; Pessia *et al.* 1996). However, coexpression of Kir5.1 with Kir4.1 produces channels that are inhibited by intracellular acidification with the pK_a_ of 7.45 (Xu *et al.* 2000). In addition to Kir4.1, Kir5.1 can also coassemble with Kir4.2 (Pearson *et al.* 1999; Pessia *et al.* 2001), although little is known about the expression of Kir4.2 in the brain.

Both Kir4.1 and Kir5.1 genes are expressed within the chemoreceptor areas of the medulla oblongata and pons, including LC, NTS and regions near the ventral surface of the medulla (Wu *et al.* 2004). As heteromeric Kir4.1–Kir5.1 channels are highly pH sensitive within the physiological range of pH changes in the brain and strongly expressed in the brainstem, we examined the role of these channels in mediating respiratory responses to chemosensory challenges using transgenic animals. A global knockout of Kir4.1 showed extreme pathology, with survival of homozygotes limited to 3 weeks postnatal (Kofuji *et al.* 2000). In contrast, mice deficient in Kir5.1 (Kir5.1^−/−^) are overtly healthy as homozygotes and show no premature mortality. Furthermore, the pH sensitivity of chemosensitive LC neurons was found to be markedly reduced in Kir5.1^−/−^ animals (D'Adamo *et al.* 2011). We therefore used adult Kir5.1^−/−^ mice to determine the role of Kir5.1 in the mechanisms responsible for the development of adaptive respiratory responses to high CO_2_ (hypercapnia) and low O_2_ (hypoxia).

## Methods

### Animals

Generation of the *Kcnj16* knockout strain (Kir5.1^−/−^) has been described elsewhere (D'Adamo *et al.* 2011), and the mice used in this study were obtained from the MRC Harwell stock (http://www.mousebook.org). These mice were on a C57Bl/6 background, were maintained as heterozygotes and were isogenic. For the experiments described here adult, (3–4 months) Kir5.1^−/−^ mice and their respective wild-type (Kir5.1^+/+^) littermates were used. All genotypes were confirmed post mortem. All experimental procedures were approved and carried out in accordance with the UK Animals (Scientific Procedures) Act (1986) and associated guidelines.

### Whole-body plethysmography

Respiratory rate (*f*_R_, in breaths per minute) and tidal volume (*V*_T_, in microlitres per gram) in conscious freely moving mice were assessed by whole-body plethysmography as described previously (Rong *et al.* 2003; Trapp *et al.* 2008). The animal was placed in a recording chamber (∼200 ml) which was flushed continuously with a humidified mixture of 79% nitrogen and 21% oxygen (unless otherwise required by the protocol) at a rate of ∼1 L min^−1^ (temperature 22–24°C). Levels of O_2_ and CO_2_ in the chamber were monitored online using a fast-response O_2_/CO_2_ analyser (Morgan Medical, Hertford, UK). The animals were allowed ∼30 min to acclimatize to the chamber at normoxia–normocapnia (21% O_2_, 79% N_2_ and <0.3% CO_2_) before measurements of baseline ventilation were taken. Hypoxia was induced by lowering the O_2_ concentration in the chamber down to 10% (with the balance being N_2_) for 5 min. Normoxic hypercapnia was induced by titrating CO_2_ into the chamber gas mixture up to a level of 3 and 6% (lowering N_2_ accordingly) for 5 min at each CO_2_ level. In a separate experiment, the animals were placed in a hyperoxic environment (>70% O_2_, with the balance being N_2_), and hypercapnia was induced 30 min later by an addition of CO_2_ up to the levels of 3 and 6% in accord with the above protocol. The measurements of the ventilatory variables were obtained during the last 2 min before exposure to the stimulus and during the 2 min period near the termination of each stimulus, when breathing had stabilized. Before and after each of the experiments, the plethysmograph was calibrated by repeated injections and withdrawal of air (0.05, 0.1 and 0.2 ml) from within the recording chamber. Changes in *f*_R_, *V*_T_ and minute ventilation (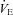; *f*_R_×*V*_T_; in millilitres per minute per gram) were averaged and expressed as means ±s.e.m.

### *In situ* brainstem–spinal cord preparation

*In situ* brainstem–spinal cord preparations (Paton, 1996) with denervated peripheral chemoreceptors were used to study the contribution of Kir channels to central respiratory chemosensitivity. In brief, Kir5.1^+/+^ and Kir5.1^−/−^ mice were injected with heparin (500 units, i.p.), deeply anaesthetized with isoflurane until loss of paw withdrawal reflex, bisected under the diaphragm, placed in cold (4°C) Ringer solution saturated with 95%O_2_–5% CO_2_, and decerebrated precollicularly. Preparations were then placed in a recording chamber and retrogradely perfused via the descending aorta with carbogenated (saturated with 95% O_2_–5% CO_2_) solution containing (mm): 124 NaCl, 26 NaHCO_3_, 3 KCl, 2 CaCl_2_, 1.25 MgSO_4_, 1.25 KH_2_PO_4_ and 10 dextrose (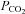 40 mmHg, pH 7.4, 32°C). To maintain oncotic pressure, Ficoll 70 (1.25%) was added. Vecuronium bromide (4 μg ml^−1^) was used to block neuromuscular transmission. When required by the experimental protocol, both vagi, aortic and carotid sinus nerves were sectioned to eliminate inputs from the peripheral chemoreceptors. Phrenic nerve activity was recorded using a suction electrode. Activity was amplified, filtered (0.1–3 kHz), rectified and integrated (50 ms time constant), relayed to a computer via a 1401 interface (CED, Cambridge, UK) and recorded using Spike2 software (CED, Cambridge, UK).

The following two stimulation protocols were used to assess respiratory responses in Kir5.1^−/−^ mice evoked by activation of central chemoreceptors. (i) Respiratory acidosis – the amount of CO_2_ bubbled through the perfusion solution was first lowered to ∼3.5% (resulting in a solution with 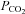 35 mmHg and pH 7.5) and then increased to 8% (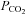 85 mmHg, pH 7.1). (ii) Isohydric hypercapnia – the preparations were perfused with a solution containing 64 mm HCO_3_^−^ with the addition of extra CO_2_ to achieve the pH level of 7.5 and 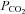 85 mmHg. In all the solutions, 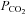 and pH values were measured using a Siemens Blood Gas Analyzer (Siemens Healthcare Diagnostics Ltd, Frimley, UK). In preparations with intact peripheral chemoreceptors, CN^−^ (0.03% w/v; 50 μl bolus) was given to activate carotid body chemoreceptors.

### *In vitro* sinus nerve recording

The function of the peripheral chemoreceptors in Kir5.1^−/−^ mice was assessed in superfused preparations of the carotid body and carotid sinus nerve as described previously (Rong *et al.* 2003; Trapp *et al.* 2008). Briefly, the animals were terminally anaesthetized with isoflurane, decapitated at the lower cervical level, and the carotid bifurcation containing the carotid body and the attached sinus nerve was dissected under a microscope and placed into a recording chamber (1 ml). The preparation was superfused with a solution containing (mm): 124 NaCl, 3 KCl, 2 CaCl_2_, 26 NaHCO_3_, 1.25 NaH_2_PO_4_, 1 MgSO_4_ and 10 d-glucose, saturated with 95% O_2_–5% CO_2_ (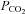 40 mmHg, pH 7.4, 37°C). Perfusion rate was 6 ml min^−1^. The sinus nerve was recorded using a suction electrode. The signal was amplified, filtered (0.2–3 kHz), relayed to a computer and recorded using 1401 interface and Spike2 software.

The following three stimulation protocols were used to assess carotid body chemosensory function. (i) Hypoxia – 3 min of superfusion of the preparation with the above solution in which O_2_ had been replaced by saturating it with 95% N_2_–5% CO_2_. (ii) Respiratory acidosis – 5 min of preparation superfusion with the solution in which extra CO_2_ had been added to increase 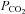 from its normal value of 40 mmHg to 80 mmHg, which is accompanied by a reduction in pH from 7.4 to 7.1. (iii) Isocapnic acidosis – the preparations were perfused with a solution containing 10 mm HCO_3_^−^ saturated with 95% O_2_–5% CO_2_ to achieve the pH level of 7.0 at normal 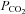 of 40 mmHg.

### Measurements of blood gases and pH

Mice were terminally anaesthetized with a mixture of ketamine (100 mg kg^−1^; i.m.) and medetomidine (250 μg kg^−1^, i.m.). A sample of arterial blood was collected from the left ventricle of the heart, and the levels of 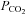, [HCO_3_^−^] and pH were immediately determined using a Siemens Blood Gas Analyzer.

### Data analysis

Recordings were processed using a 1401 interface and analysed using Spike2 software. All the data are reported as means ±s.e.m. Comparisons between experimental groups were made using unpaired Student's *t* test or ANOVA followed by the Tukey–Kramer *post hoc* test, as appropriate. A value of *P* < 0.05 was considered to be significant.

## Results

### Ventilatory responses to hypoxia and hypercapnia in Kir5.1^−/−^ mice

**Figure 1:**
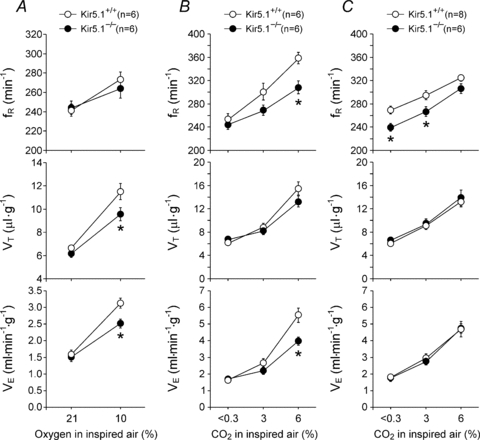
Reduced ventilatory responses to hypoxia and normoxic hypercapnia in Kir5.1^−/−^ mice*A*, ventilatory responses to hypoxia (10% O_2_ in the inspired air) in conscious Kir5.1-deficient mice (Kir5.1^−/−^) and their wild-type counterparts (Kir5.1^+/+^). *B*, ventilatory responses to varying levels of normoxic hypercapnia in Kir5.1^−/−^ and Kir5.1^+/+^ mice. *C*, ventilatory responses to varying levels of hyperoxic (>70% O_2_ in the inspired air) hypercapnia in Kir5.1^−/−^ and Kir5.1^+/+^ mice. Data are presented as means ±s.e.m. Numbers in parentheses indicate sample sizes. Abbreviations: *f*_R_, respiratory rate; *V*_T_, tidal volume; and 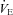, minute ventilation (*f*_R_×*V*_T_). *Significantly different (*P* < 0.05) from Kir5.1^+/+^ mice.

In resting conditions (normoxia–normocapnia), ventilation was similar in Kir5.1^−/−^ mice (1.51 ± 0.07 ml min^−1^ g^−1^, *n* = 6) and their wild-type counterparts (1.59 ± 0.04 ml min^−1^ g^−1^, *n* = 6; *P* = 0.37). When exposed to hypoxic conditions (10% O_2_ in the inspired gas mixture), both Kir5.1^−/−^ and wild-type mice showed an increased rate (*f*_R_) and depth of breathing (*V*_T_) and, therefore, an increased minute ventilation (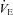; Fig. 1*A*). However, the ventilatory response to hypoxia was significantly smaller in Kir5.1^−/−^ animals (Fig. 1*A*). In 10% O_2_, Kir5.1^−/−^ animals had a 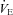 of 2.52 ± 0.12 ml min^−1^ g^−1^ (*n* = 6), whereas in the wild-type animals 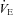 was 3.12 ± 0.15 ml min^−1^ g^−1^ (*n* = 6, *P* = 0.010; ANOVA). This difference arose from a significantly smaller *V*_T_ (but not *f*_R_) increase in response to hypoxia in Kir5.1^−/−^ animals (Fig. 1*A*)

The Kir5.1^−/−^ mice also displayed reduced ventilatory responses to increased levels of inspired CO_2_. Respiratory activities were similar in Kir5.1^−/−^ and wild-type mice in an atmosphere containing 3% CO_2_ (Fig. 1*B*). However, with 6% CO_2_, 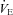 in Kir5.1^−/−^ mice increased to 4.04 ± 0.15 ml min^−1^ g^−1^ (*n* = 6), while in the wild-type animals in the same conditions 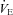 was elevated to 5.55 ± 0.43 ml min^−1^ g^−1^ (*n* = 6, *P* = 0.017; ANOVA; Fig. 1*B*). In contrast to the respiratory responses evoked by hypoxia, this difference in 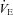 arose from a significantly smaller CO_2_-induced increase in respiratory rate (but not *V*_T_) in Kir5.1^−/−^ mice (Fig. 1*B*). In hyperoxic conditions (>70% O_2_), the resting *f*_R_ was significantly higher in wild-type mice (Fig. 1*C*); however, baseline 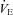 and the overall ventilatory response to hyperoxic hypercapnia was similar in Kir5.1^−/−^ animals (*n* = 6) and their wild-type counterparts (*n* = 8; Fig. 1*C*).

### Carotid body function in Kir5.1^−/−^ mice

**Figure 2:**
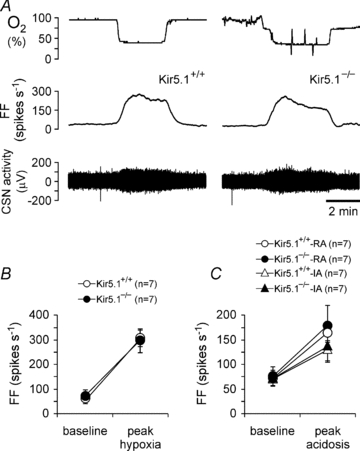
Normal carotid body function in Kir5.1^−/−^ mice*A*, representative raw data showing hypoxia-evoked increases in the carotid sinus nerve (CSN) chemoafferent discharge recorded in the *in vitro* superfused carotid body–carotid sinus nerve preparations taken from the Kir5.1-deficient mice (Kir5.1^−/−^), and their wild-type counterparts (Kir5.1^+/+^). ‘O_2_ trace’ depicts changes in oxygen concentration in the incubation chamber. *B* and *C*, summary data of the mean resting and peak hypoxia- and acidification-induced increases in the discharge frequency of the carotid sinus nerve in preparations taken from Kir5.1^−/−^ and Kir5.1^+/+^ mice. Abbreviations: RA, respiratory acidosis stimulus; IA, isocapnic acidosis stimulus (see main text for details); and FF, discharge frequency. Data are presented as means ±s.e.m. Numbers in parentheses indicate sample sizes.

The reduced hypoxic ventilatory response suggested that the function of the peripheral chemosensors may be compromised in Kir5.1^−/−^ mice. In order to test this hypothesis, we recorded chemoafferent activity of the carotid sinus nerve in an *in vitro* superfused carotid body–carotid sinus nerve preparations taken from Kir5.1^−/−^ mice and their wild-type counterparts. In baseline conditions, carotid sinus nerve chemoafferent discharge was similar in Kir5.1^−/−^ and control mice (Fig. 2). As expected, hypoxic stimulation evoked marked activation of the carotid chemoreceptors (Fig. 2*A* and *B*). However, the magnitude of the hypoxia-evoked carotid sinus nerve chemoafferent responses was similar in Kir5.1^−/−^ mice (increase in sinus nerve discharge from 76 ± 20 spikes s^−1^ to a peak of 295 ± 45 spikes s^−1^, *n* = 7) and control animals (increase from 64 ± 16 spikes s^−1^ to a peak of 307 ± 35 spikes s^−1^, *n* = 7, *P* = 0.8; Fig. 2*A* and *B*).

An *in vitro* analogue of respiratory acidosis (decrease in pH triggered by an increase in 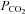 at constant [HCO_3_^−^]) activated carotid body chemosensors, albeit to a lesser degree compared with that during hypoxia (Fig. 2*C*). Chemoafferent responses evoked by respiratory acidosis were identical in Kir5.1^−/−^ (peak discharge 179 ± 40 spikes s^−1^, *n* = 7) and wild-type animals (peak discharge 164 ± 19 spikes s^−1^, *n* = 7, *P* = 0.7; Fig. 2*C*). Likewise, increases in the carotid sinus nerve discharge evoked by isocapnic acidosis (decrease in pH by lowering [HCO_3_^−^] at constant 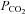) were not affected by Kir5.1 deficiency (Fig. 2*C*). Interestingly, peak increases in chemoafferent discharges triggered in wild-type preparations by respiratory acidosis (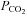 80 mmHg, pH 7.1, [HCO_3_^−^] 26 mm) were similar to that evoked by isocapnic acidosis (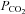 40 mmHg, pH 7.0, [HCO_3_^−^] 10 mm; Fig. 2*C*).

### Central respiratory chemosensitivity in Kir5.1^−/−^ mice

**Figure 3:**
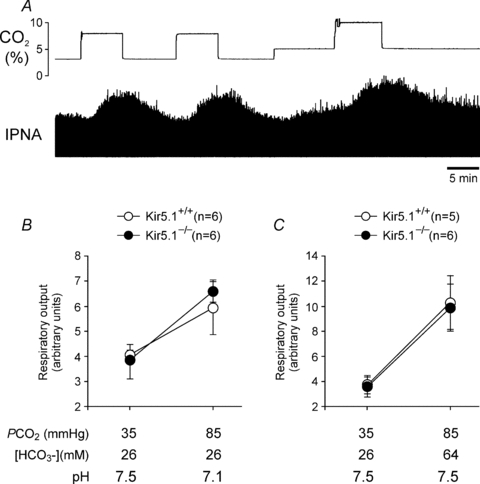
Normal central respiratory chemosensitivity in Kir5.1^−/−^ mice*A*, raw data showing time-condensed record of hypercapnia-evoked changes in integrated phrenic nerve activity (IPNA) of a wild-type *in situ* brainstem–spinal cord preparation with denervated peripheral chemoreceptors. ‘CO_2_ trace’ depicts changes in CO_2_ concentration of the gas mixture used to saturate the perfusion solution, hence there is a lag between changes in CO_2_ and resultant respiratory responses. *B* and *C*, summary data of changes in minute respiratory output (phrenic amplitude × respiratory rate) in response to respiratory acidosis or isohydric hypercapnia, respectively, in preparations of Kir5.1-deficient mice (Kir5.1^−/−^) and their wild-type counterparts (Kir5.1^+/+^). Data are presented as means ±s.e.m. Numbers in parentheses indicate sample sizes.

The data described in the previous subsection indicated that Kir5.1^−/−^ deficiency does not appear to have an effect on the carotid body function. To examine the role of Kir5.1 in central respiratory chemosensitivity, the next series of experiments was conducted in the *in situ* brainstem–spinal cord preparation with denervated peripheral chemoreceptors. Phrenic nerve activity in baseline conditions was similar in the *in situ* brainstem–spinal cord preparations of Kir5.1^−/−^ mice (*n* = 12) and their wild-type counterparts (*n* = 11, *P* = 0.1). Respiratory acidosis (decrease in pH triggered by an increase in 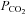 at constant [HCO_3_^−^], see Methods) evoked similar increases in respiratory activity in preparations of Kir5.1^−/−^ and wild-type mice with denervated peripheral chemoreceptors (Fig. 3*A* and *B*). Respiratory responses to isohydric hypercapnia (increase in 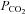 at constant pH and elevated [HCO_3_^−^]) were also unaffected by Kir5.1 deficiency (Fig. 3*C*).

### Transmission of the peripheral chemosensory stimuli in Kir5.1^−/−^ mice

**Figure 4:**
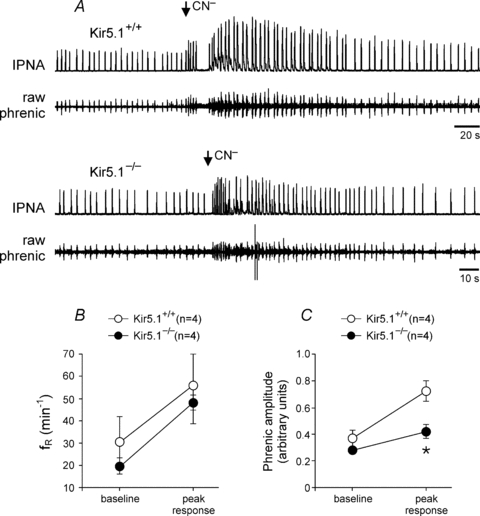
Compromised transmission of the peripheral chemosensory stimuli to the respiratory network in Kir5.1^−/−^ mice*A*, representative raw data showing changes in phrenic nerve frequency and amplitude evoked by stimulation of the peripheral chemoreceptors with cyanide (0.03% w/v; 50 μl bolus) in the *in situ* brainstem–spinal cord preparations of Kir5.1-deficient mice (Kir5.1^−/−^) and their wild-type counterparts (Kir5.1^+/+^). Also shown are summary data of mean peak CN^−^-induced increases in respiratory frequency (*f*_R_; *B*) and phrenic amplitude (*C*) in preparations of Kir5.1^−/−^ and Kir5.1^+/+^ mice. Data are presented as means ±s.e.m. Numbers in parentheses indicate sample sizes. *Significantly different (*P* < 0.05) from Kir5.1^+/+^ mice.

Reduced ventilatory responses to hypoxia and normoxic hypercapnia and apparently normal peripheral and central respiratory chemoreceptor function suggested that the relay of the peripheral chemosensory stimuli is compromised in Kir5.1^−/−^ mice. To test this hypothesis, *in situ* brainstem–spinal cord preparations with intact peripheral chemoreceptors were used. Activation of the carotid body chemoreceptors in preparations from Kir5.1^−/−^ mice following bolus application of CN^−^ resulted in a similar frequency response (Fig. 4*B*), but significantly smaller increases in phrenic nerve amplitude in comparison to that in wild-type preparations (increase by 47 *versus* 95%, *P* = 0.019, ANOVA; Fig. 4*A* and *C*)

### Arterial pH, 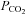 and [HCO_3_^−^] in Kir5.1^−/−^ mice

Kir5.1 has been found in the kidney (Tucker *et al.* 2000) and thus might influence the acid–base balance of the organism. In agreement with this hypothesis, adult Kir5.1^−/−^-deficient mice were found to have a profound metabolic acidosis, with arterial blood pH of 7.26 ± 0.03 (*versus* 7.38 ± 0.01 in the wild-type animals, *P* = 0.003, unpaired Student's *t* test), 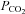 of 41.6 ± 3.9 mmHg (*versus* 44.6 ± 2.6 mmHg in the wild-type animals, *P* = 0.68, unpaired Student's *t* test) and [HCO_3_^−^] of 18.1 ± 1.6 mm (*versus* 25.6 ± 0.2 mm in the wild-type animals, *P* = 0.005, *n* = 6, unpaired Student's *t* test).

## Discussion

This study examined the potential contribution of Kir5.1 to the chemosensory control of breathing. Kir5.1 was an obvious target, because its coexpression with Kir4.1 produces K^+^ channels which are inhibited by decreases in intracellular pH within the physiological range (Xu *et al.* 2000). Although other members of the Kir family, including homomeric Kir4.1, are pH sensitive, their sensitivity lays well outside the physiological range of pH changes in the blood or brain. If pH-sensitive heteromeric Kir4.1–Kir5.1 channels are functionally important, then in Kir5.1 knockout animals the homomeric Kir4.1 channels will remain but the resultant Kir conductance will not be pH sensitive within the physiological range.

Reduced ventilatory responses to both hypoxia and normoxic hypercapnia and similar CO_2_-evoked increases in breathing in conditions of hyperoxia initially suggested that the function of the peripheral chemosensors may be compromised in Kir5.1^−/−^ mice. Indeed, expression of both Kir4.1 and Kir5.1 by the peripheral chemoreceptors has been previously demonstrated (Yamamoto *et al.* 2008*a*). Chemosensitive glomus cells of the carotid body as well as some of the petrosal ganglion cells have been found to express Kir4.1 and Kir5.1 (Yamamoto *et al.* 2008*a*). However, our results suggest that these channels are likely to be dispensable for chemosensory function of the carotid body, since increases in the carotid sinus nerve chemoafferent discharge evoked by decreases in pH within the physiological range were not affected by deletion of Kir5.1. We also noted that the peak chemoafferent response triggered in wild-type as well as Kir5.1^−/−^ carotid body–carotid sinus nerve preparations by respiratory acidosis were similar in amplitude to that evoked by isocapnic acidosis (Fig. 2*C*). Isocapnic acidosis would be expected to evoke smaller/delayed changes in intracellular pH, further weakening the case for Kir channels to be involved and intracellular pH to be the prime stimulus responsible for activation of the peripheral chemosensors in response to hypercapnia. Indeed, according to our previous observations the carotid body chemoafferent responses to increases in 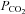/[H^+^] are almost entirely mediated by homomeric TASK-1 or heteromeric TASK-1–TASK-3 channels (Trapp *et al.* 2008), which are predominantly sensitive to changes in extracellular pH.

The involvement of heteromeric Kir4.1–Kir5.1 channels in the central respiratory chemosensitivity to changes in 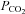/[H^+^] has been suggested previously (Jiang *et al.* 2001) on the basis of their high sensitivity to changes in intracellular pH (Xu *et al.* 2000) and their abundant coexpression in the brainstem areas known to contain functional respiratory chemoreceptors (Wu *et al.* 2004). However, patterns of Kir4.1 and Kir5.1 expression in these regions are complex, with clear overlapping expression observed in some studies (Wu *et al.* 2004; Zhang *et al.* 2010), but not in the others (Yamamoto *et al.* 2008*b*). The study by Yamamoto *et al.* (2008*b*) also demonstrated strong expression of Kir4.1 in the brainstem glial cells. Intriguingly, Wenker *et al.* (2010) reported that inhibition of Kir4.1–Kir5.1-like channels may be responsible for pH-evoked depolarization of glial cells residing within the anatomical region of the RTN, which is one of the key central chemosensitive sites. However, our recent study of the role of astrocytes in central respiratory chemosensitivity (Gourine *et al.* 2010) argues against changes in astrocytic membrane potential as the key event in the central chemosensory transduction mechanism.

Regardless of any potential role for glial cells and the uncertainty of precisely which brainstem neurons may coexpress Kir4.1 and Kir5.1, we still observe a clear respiratory phenotype in Kir5.1^−/−^ mice. However, the data obtained suggest that Kir channels are unlikely to be essential for functional central respiratory sensitivity to changes in 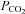/[H^+^]. Interestingly, using the same Kir5.1^−/−^ mice, D'Adamo *et al.* (2011) demonstrated that Kir5.1 contributes to pH-evoked responses of one functional population of central chemosensitive neurones in the LC. Together, these data and observations reported herein suggest that LC neuronal input may not be essential for the respiratory responses evoked by central actions of CO_2_ in adult mice.

The metabolic acidosis observed in the Kir5.1^−/−^ mice is consistent with the expression of Kir5.1 in the kidney (Tucker *et al.* 2000). Interestingly, development of metabolic acidosis was previously reported after genetic ablation of another notable kidney K^+^ channel, TASK-2 (Warth *et al.* 2004). Heteromeric Kir4.1–Kir5.1 channels have been identified in the basolateral membrane of distal tubular epithelia and are thought to be involved in the recycling of K^+^ across this membrane and H^+^/K^+^ homeostasis (Lourdel *et al.* 2002; Lachheb *et al.* 2008). Detailed investigation of renal function in Kir5.1-deficient mice is beyond the scope of the present study; however, the effect of persistent metabolic acidosis on the chemosensory control of breathing warrants further discussion.

The data obtained in the present study reveal markedly lower levels of the key homeostatic constants, pH and [HCO_3_^−^], in the arterial blood of adult Kir5.1^−/−^ mice. This would be expected to have a stimulatory effect on central and peripheral chemoreceptors; however, despite profound metabolic acidosis the resting ventilation in Kir5.1^−/−^ animals was similar to that in their wild-type counterparts (similar to observations by Gestreau *et al.* 2010 in TASK-2 knockout mice, which in conditions of metabolic acidosis also display normal respiratory activity at rest). This could be explained by compensatory reduction in peripheral chemoreceptor sensitivity, but the data obtained in our *in vitro* experiments demonstrate that at a given level of 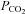, pH and [HCO_3_^−^] the Kir5.1^−/−^ and Kir5.1^+/+^ preparations exhibit the same level of carotid sinus nerve chemoafferent discharge. Moreover, chemoafferent responses to isohydric hypercapnia (mimicking metabolic acidosis) are similar in Kir5.1^−/−^ and wild-type animals. By extension, these data suggest that in conditions of continuing metabolic acidosis associated with Kir5.1 deficiency the resting carotid chemoafferent activity should be higher in Kir5.1^−/−^ mice; however, this is not translated into facilitated ventilatory activity. Indeed, selective CN^−^-evoked activation of the carotid body chemoreceptors in the *in situ* preparations of Kir5.1^−/−^ mice was found to result in a significantly smaller increase in phrenic nerve amplitude in comparison to that of wild-type preparations. This suggests that the relay of peripheral chemosensory information to the CNS is compromised by Kir5.1 deficiency; however, the exact nature of this compensatory mechanism as yet remains unknown.

The results obtained using a global gene knockout model have to be considered with caution, since the loss of a gene may be compensated for by increased expression of one or several other genes. In the latter case, this approach may not reveal the true functional role of a protein in question. A recent study by D'Adamo *et al.* (2011) demonstrated ablated pH-evoked responses of LC neurones in Kir5.1^−/−^ animals and suggested that the loss of the Kir5.1 may not be fully compensated for, at least in the LC.

In summary, the data obtained in the present study demonstrate that although deletion of Kir5.1 in mice results in a clear respiratory phenotype, it appears that the loss of Kir5.1 does not directly affect the function of either central or peripheral respiratory chemoreceptors. Despite a profound metabolic acidosis, the resting ventilation in Kir5.1^−/−^ mice is similar to that of the wild-type control animals. The ventilatory responses to systemic hypoxia and hypercapnia are reduced, as transmission of the signals from the peripheral chemoreceptors to the CNS appear to be compromised. We suggest that this compensatory modulation of the peripheral chemosensory inputs develops in order to counteract the effect of continuing metabolic acidosis on the activity of the peripheral chemoreceptors.
